# COMPLICATIONS AND MIDTERM OUTCOMES OF HEMIARTHROPLASTY IN HEMODIALYSIS PATIENTS

**DOI:** 10.1590/1413-785220172505167473

**Published:** 2017

**Authors:** AHMET SALDUZ, GÖKHAN POLAT, TURGUT AKGÜL, OMER NACI ERGIN, KORAY ŞAHIN, ÖNDER YAZICIOĞLU

**Affiliations:** 1. Department of Orthopedics and Traumatology, Istanbul University Istanbul Medical Faculty, Istanbul, Turkey.

**Keywords:** Femoral neck fractures/complications, Kidney failure, chronic/complications, Hemiarthroplasty, Hemodialysis, Mortality

## Abstract

**Objective:**

The aim of this study was to evaluate the functional results, complications, and morbidity and mortality rates in patients with end-stage chronic renal failure (ESCRF) with collum femoris fractures who were treated with hemiarthroplasty.

**Methods:**

From 2005 to 2013, patients with ESCRF admitted to our hospital with collum femoris fracture and treated with hemiarthroplasty were retrospectively evaluated, and 44 hips in 42 patients were included in the study. Duration of hospital stay, bleeding, complications, morbidity and mortality were recorded for each patient. At the last control evaluation, patients were assessed via pelvis x-ray and functional status according to Harris Hip Score (HHS).

**Results:**

Patients required a mean 2.7 units of erythrocyte suspension. Mean hospital stay was 19.74 days. The most common complication was bleeding. The complication rate was 38.1%; mortality rate at first-year follow-up was 42.8%, and mean HHS was 74.5.

**Conclusion:**

Collum femoris fractures are more common in ESCRF patients due to metabolic bone disease, and these patients had many comorbidities which may exacerbate high complication and mortality rates. Orthopedic surgeons should consider these higher complication rates and inform patients about the consequences of this treatment. **Level of Evidence IV, Case Series.**

## RESUMO

**Objetivo:**

O objetivo deste estudo foi avaliar os resultados funcionais, as complicações e as taxas de morbidade e mortalidade em pacientes com insuficiência renal crônica em estágio terminal (IRCT) com fraturas do colo do fêmur tratados com hemiartroplastia.

**Métodos:**

De 2005 a 2013, pacientes com IRCT internados em nosso hospital com fratura do colo do fêmur e tratados com hemiartroplastia foram avaliados retrospectivamente, e 44 quadris em 42 pacientes foram incluídos no estudo. Durante a estadia hospitalar, hemorragia, complicações, morbidade e mortalidade foram registradas para cada paciente. Na última avaliação de controle, os pacientes foram examinados com radiografias da pelve e quanto ao estado funcional, de acordo com o Harris Hip Score (HHS).

**Resultados:**

Os pacientes precisaram em média 2,7 unidades de suspensão de eritrócitos. A estadia hospitalar média foi 19,74 dias. A complicação mais comum foi hemorragia. A taxa de complicações foi 38,1%; a taxa de mortalidade no primeiro ano de acompanhamento foi 42,8% e o HHS médio foi 74,5.

**Conclusão:**

As fraturas de colo do fêmur são mais comuns em pacientes com IRCT, em decorrência da doença óssea metabólica, e esses pacientes apresentam muitas comorbidades que podem exacerbar as altas taxas de complicação e mortalidade. Os cirurgiões ortopédicos precisam considerar esses altos índices de complicações e informar os pacientes sobre as consequências desse tratamento. **Nível de Evidência IV, Série de Casos.**

## INTRODUCTION

Patients with end-stage renal failure have metabolic bone disease related to hemodialysis and comorbidities with renal failure such as low albumin levels, changes in calcium and phosphate levels, and high parathyroid hormone levels.^1^ ^-^ ^3^ In hemodialysis patients, this metabolic bone disease brings with it 4 to 5 times the risk of fatigue or traumatic fractures, particularly collum femoris fractures.^1^ ^,^ ^3^ ^,^ ^4^ In addition to the high risk of the collum femoris fractures in this group of patients, this metabolic disease causes problems in osteosynthesis of these fractures due to lower bone mineral density (BMD).^3^ ^-^ ^7^ Therefore, in treating collum femoris fractures many authors have cited arthroplasty due to the high risk of failed osteosynthesis, non-union, avascular necrosis, and the need for revision surgery.^5^ ^-^ ^7^

On the other hand, these are high-risk patients for surgical procedures related to comorbidities such as diabetes mellitus, coronary artery disease, and peripheral arterial diseases.^8^ ^,^ ^9^ Although arthroplasty seems the most sensible treatment option for problematic patients, the rates of complication, prosthesis survival, morbidity, and mortality for these patients are not well known because the literature on these topics is limited.^6^ ^,^ ^7^

The aim of this study was to evaluate the early and midterm functional results and rates of complications, prosthesis survival, morbidity, and mortality in patients with chronic renal failure who had collum femoris fractures and were treated with hemiarthroplasty. 

## MATERIALS AND METHODS

This retrospective study included 44 hips in 42 patients with chronic renal failure admitted to our clinic for collum femoris fractures and treated with hemiarthroplasty between 2005 and 2013. The surgical reports were retrieved from a computerized database, and three surgeons reviewed the patient charts for exclusion and inclusion criteria.

Mean patient age was 63.5 years (range: 49-95 years, standard deviation: 9.47) with mean follow-up of 52.3 months (range: 6-192, standard deviation: 19.63). Patients who underwent revision arthroplasty due to failed osteosynthesis, non-union, and avascular necrosis were excluded. Furthermore, all the patients included in the study underwent dialysis to treat end-stage renal failure. 

Duration of hospital stay, postoperative bleeding, complications, morbidities, and mortality for the patients were recorded in the hospital’s computerized database and in each patient’s medical records. Two surgeons made a final control evaluation of the surviving patients and their functional status using the Harris hip score (HHS); patients were evaluated for prosthesis survival via anteroposterior (AP) pelvic x-ray and AP and lateral x-rays of the operated hip. 

Detailed information on the surgical interventions was provided to all patients, and all patients signed an informed consent form for the surgical technique performed. This study was approved by the institutional review board (2014/1340).

### Surgical technique

The posterior approach was used in all surgeries, which were performed by four surgeons. Patients were placed in lateral decubitus position. After the fractured head was exposed and removed, the femoral neck was reshaped and the appropriate polished, cemented femoral stem (Spectron, Smith & Nephew) was inserted. An appropriately-sized unipolar or bipolar head was implanted, and then reduction and stability control were performed. Posterior soft tissues were then repaired, and the fascia, subcutaneous tissue, and skin were closed following normal procedure. 

After admittance, all patients received antiembolic socks and low-molecular-weight heparin (4000 anti-Xa IU/0.4 ml) as prophylaxis against deep-vein thrombosis. All patients were seen by nephrology specialists, and surgery was performed according to suggestions from these specialists. All patients received hemodialysis prior to the day of surgery and preoperative values for potassium and creatinine were closely monitored. All patients received prophylactic antibiotic therapy (first-generation cephalosporin 3 mg/kg) before surgery, which was maintained until the second day after the procedure.

Postoperative rehabilitation was planned according each individual patient. Patients walked with the aid of a walker the first day after the procedure if their general condition permitted this activity. Mobilization was postponed for patients requiring intensive care unit maintenance after surgery until they were admitted to the orthopedic service. Sutures were removed on the 15th day post-procedure and patients were followed at intervals of 6 weeks, 3 months, 6 months, and 1 year after surgery. 

### Statistical analysis

SPSS for Windows v12.0 software (SPSS Inc., Chicago, IL, USA) was used for the statistical analysis. In quantitative comparisons, data were assessed using Student’s t-test and paired sample t-tests. For qualitative comparisons, data were assessed using the chi-square and Fisher’s exact chi-square tests. Statistical significance was accepted at a 95% confidence interval and for p-values less than 0.05. 

## RESULTS

The mean age of the 42 patients (44 hips) was 63.5 years (range: 49-95 years, standard deviation: 9.47) with mean follow-up of 52.3 months (range: 6-192, standard deviation: 19.63). Twenty-nine patients were female and 13 were male. (p=0.083) The left hip was affected in 24 patients, and the right in 20 patients. Two patients had collum femoris fractures at different times and were treated with hemiarthroplasty. 

A cemented femoral stem (Spectron, Smith & Nephew) was selected for all patients; 18 received a bipolar femoral head and 26 received a unipolar femoral head. There was no statistical difference between bipolar and unipolar heads with regard to patient hip dislocation rates. (p=0.149) 

The average duration of hemodialysis treatment prior to fracture occurrence was 10.3 years (range: 4 months-25 years). Mean preoperative hemoglobin and hematocrit levels were 7.96 mg/dl and 22.4%, respectively. (range: 6.1 mg/dl-9.7 mg/dl and 16.3%-28.5%, standard deviation: 1.31 mg/dl and 3.01%). Mean postoperative blood loss was 900 cc (range: 200 cc-3000 cc, standard deviation: 250 cc) and mean transfusion volume was 2.7 units of erythrocyte suspensions (range: 2-8, standard deviation: 1.47). Mean duration of hospital stay was 19.74 days (range: 8-120 days, standard deviation: 4.68).

The most common complication (8 patients) was bleeding in our patient series. The other complications were early prosthetic infection (2 patients), hip dislocation (1 patient), myocardial infarction during hospital stay (2 patients), pulmonary embolism (1 patient), epidural cranial bleeding (1 patient), and sepsis due to cholecystitis during hospital stay (1 patient). (Table 1) The total complication rate in our patient sample was 38.1%. No correlation was found between complications and average duration of hemodialysis. (p=0.092) 

Five patients died during hospitalization as a result of myocardial infarction (2 patients), pulmonary embolism (1 patient), sepsis (1 patient), and congestive heart failure in intensive care unit (1 patient). The mortality rate of the patients at the 1-year follow-up was 42.8% (18/42). At the last control evaluation, the mortality rate was 59.5% (25/42). 

**Table 1:** Complications.

Complications:	Number of patients
Bleeding	8
Prosthetic infection	2
Hip dislocation	1
Pulmonary embolism	1
Myocardial infarction	2
Epidural cranial bleeding	1
Sepsis	1

**Figure 1:**
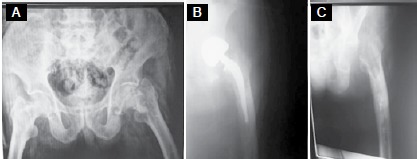
80-year-old male patient with left collum femoris fracture. (A) Preoperative AP view of pelvis; (B) Postoperative AP view of left hip; (C) AP view of left hip after implant removal due to ongoing periprosthetic infection.

**Figure 2:**
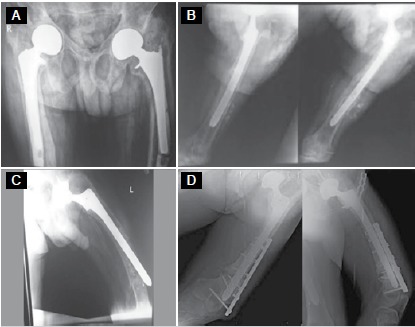
65-year-old male patient. (A) AP view of pelvis showing left femoral component loosening at 7-year follow-up. (B) AP and lateral views of left hip after revision surgery. (C) AP view of left femur showing periprosthetic femoral fracture; (D) AP view of left femur after osteosynthesis of the fracture.

In the assessment of prosthesis survival, 2 patients required implant removal due to ongoing infection after debridement treatment, and did not undergo a revision procedure due to poor medical conditions. (Figure 1) One patient had an acetabular protrusion in the second year post-procedure and underwent a revision procedure entailing total hip arthroplasty. One patient had loosening of the femoral component and underwent a revision procedure in the seventh year of follow-up; this same patient had a periprosthetic femoral fracture after one year and was treated with osteosynthesis. (Figure 2) There were 17 living patients at the last follow-up, and the mean HHS of these patients was 74.5 (range: 43-85, standard deviation: 6.63). 

## DISCUSSION

Treatment of collum femoris fractures in patients with chronic renal failure is still challenging because of the low bone mineral density in these patients.^1^ ^,^ ^2^ ^,^ ^5^ ^,^ ^6^ Although the metabolic problems in these patients do not prevent fracture union, the risk of osteosynthesis failure is usually higher due to their low bone mineral density, and the literature favors arthroplasty, especially in middle-aged and older patients.^5^ ^,^ ^6^ Although arthroplasty is a more sensible treatment option in comparison to osteosynthesis, the complications and outcomes of arthroplasty are not well known in this special group of patients because the literature on this topic is limited.^6^ ^,^ ^10^ Our study evaluated the midterm functional results, complications, and morbidity and mortality rates in patients with end-stage chronic renal failure who had collum femoris fractures and were treated with hemiarthroplasty.

There are some studies in the literature that investigate the results of hip fracture treatment in patients with chronic renal failure.^10^ ^-^ ^12^ Karaeminoğulları et al.^10^ retrospectively evaluated 29 patients with renal failure in three groups (osteosynthesis of intertrochanteric fractures, arthroplasty of collum femoris fractures, and osteosynthesis of collum femoris fractures) and suggested arthroplasty instead of osteosynthesis in collum femoris fractures in chronic renal failure patients because of high complication rates in these patients.^10^ These authors reported 1 complication in 8 arthroplasty operations (12.5%).^10^ Another study from Poland reported hemiarthroplasty results in 12 patients with chronic renal failure (mean age: 51 years) and did not observe any serious complications, other than one acetabular protrusion 20 months after surgery.^11^ In our study we had a more homogeneous group of 42 patients and 44 hips. In comparison to these previous studies, our complication rate of 38.1% was notably higher. We also had 2 cases which required revision surgery, and at the last control evaluation, prosthesis survival of our living patients was 76.4% with mean follow-up of 52.3 months. 

Bleeding was the most common complication in our patients, and 2 patients required secondary surgery for hematoma evacuation. This complication may result from the use of heparin to prevent coagulation during hemodialysis. On the other hand, these patients had coagulopathy problems, and in addition to the risk of hip fracture these patients are more prone to vascular complications. Although all patients received prophylaxis with low-molecular-weight heparin, 2 patients had myocardial infarction and 1 patient had a pulmonary embolism. Infection is another common complication, and prosthetic infection varies from 0 to 19% between different patient series.^6^ ^,^ ^12^ This high risk of periprosthetic infection may be related to mismanagement of hematoma in the postoperative period. In our series, we had 2 periprosthetic infections (4.7%). Although these infections were diagnosed in the early postoperative period, both patients required implant removal due to ongoing infection after debridement treatment and did not receive revision surgery because of their poor medical condition.

In another study, Klein et al.^13^ reported treatment results for 9 hips in 8 patients (5 osteosynthesis, 4 arthroplasty). In this small series no wound infections, thromboembolic events, or hemorrhagic complications were reported, but these authors did note a 38% mortality rate in the first year after surgery. The mortality rate for senile osteoporotic hip fractures without chronic renal failure has been reported in different series as 11-24%.^14^ In our patients, the first-year mortality rate was 42.8%. This difference may be associated with comorbidities such as cardiovascular, endocrine, gastrointestinal, and infectious diseases related to chronic renal failure. 

Sakabe et al.^15^ evaluated life expectancy and function after collum femoris fractures in a total of 71 hemodialysis patients; 13 were treated non-surgically, 34 received hemiarthroplasty and 24 received internal fixation. In 34 patients who underwent hemiarthroplasty, these authors reported 14.7% dislocation of prosthesis, 8.6% bleeding, and 1.7% infection. The entire patient group in this study underwent evaluation for returning to daily living activities, and the authors reported that over 50% of the patients had returned to these activities at 1 year after surgery.^15^ Complication rates for this patient series were similar to those found in this present study. In comparison to this study, we evaluated functional status in 17 living patients at the last follow-up and found a mean HHS score of 74.5 (range 43-85).

The main limitations of our study were its retrospective nature and the lack of a matched control group comprised of non-hemodialysis patients with collum femoris fractures. However, our patient series is one of the largest in the literature and to our knowledge is the only study evaluating the complications and midterm outcomes of hemiarthroplasty treatment alone in this special group of patients. 

## CONCLUSION

Collum femoris fractures are more common in hemodialysis patients because of metabolic bone disease in chronic renal failure. In addition to low bone mineral density, these patients had many comorbidities such as coronary artery disease and coagulopathies, and these problems may exacerbate the high complication rates (38.1%) and high first-year mortality rates (42.8%) in these patients. Orthopedic surgeons should consider this high complication rate and inform patients and their families about the consequences of this treatment. 
